# Missing Puzzle Pieces in Dementia Research: HCN Channels and Theta Oscillations

**DOI:** 10.14336/AD.2023.0607

**Published:** 2024-02-01

**Authors:** Paulina Kazmierska-Grebowska, Maciej M. Jankowski, M. Bruce MacIver

**Affiliations:** ^1^Department of Neurobiology, Faculty of Biology and Environmental Protection, University of Lodz, Poland.; ^2^Edmond and Lily Safra Center for Brain Sciences, The Hebrew University of Jerusalem, Jerusalem, Israel.; ^3^BioTechMed Center, Multimedia Systems Department, Faculty of Electronics, Telecommunications, and Informatics, Gdansk University of Technology, Gdansk, Poland.Telecommunications and Informatics, Gdansk University of Technology, Gdansk, Poland.; ^4^Department of Anesthesiology, Perioperative and Pain Medicine, Stanford University School of of Medicine, Stanford University, CA, USA.

**Keywords:** Alzheimer's disease, dementia, lamotrigine, HCN channels, Ih current, theta oscillations, memory, EEG

## Abstract

Increasing evidence indicates a role of hyperpolarization activated cation (HCN) channels in controlling the resting membrane potential, pacemaker activity, memory formation, sleep, and arousal. Their disfunction may be associated with the development of epilepsy and age-related memory decline. Neuronal hyperexcitability involved in epileptogenesis and EEG desynchronization occur in the course of dementia in human Alzheimer’s Disease (AD) and animal models, nevertheless the underlying ionic and cellular mechanisms of these effects are not well understood. Some suggest that theta rhythms involved in memory formation could be used as a marker of memory disturbances in the course of neurogenerative diseases, including AD. This review focusses on the interplay between hyperpolarization HCN channels, theta oscillations, memory formation and their role(s) in dementias, including AD. While individually, each of these factors have been linked to each other with strong supportive evidence, we hope here to expand this linkage to a more inclusive picture. Thus, HCN channels could provide a molecular target for developing new therapeutic agents for preventing and/or treating dementia.

## INTRODUCTION

This review examines the function of hyperpolarization activated cation (HCN) channels in the modulation of neuronal excitability, EEG theta band synchronization and memory formation. It gives recent insights into disturbances in HCN-dependent inward cationic current physiology and its’ contribution to possible development of dementias, such as Alzheimer's disease (AD). HCN channels generate hyperpolarization activated cationic currents (I*h)* seen in many types of neurons. Recent evidence has suggested that I*h* is involved in the determination of resting membrane potential, pacemaker activity, membrane potential oscillations (MPOs), synaptic plasticity [1-8], learning and memory, sleep and arousal [3, 6, 9-11], epilepsy and seizures [12-25], age-related memory decline [11, 26], and more [3, 6]. HCN channels are considered suitable drug targets for several central nervous system pathologies such epilepsy, pain, depression, and Parkinson’s Disease [27], in addition to dementias. Our recent studies have revealed that Lamotrigine (LTG), a non-specific modulator of HCN channels, is able to decrease neuronal excitability and reduce GABA-mediated synaptic inhibition in hippocampal slices. Additionally, we have found that LTG can also modulate theta activity in rat hippocampus *in vivo* [28]. There is a body of evidence that neuronal hyperexcitability, epilepsy and EEG desynchronization occur in the course of dementia in human AD and animal models of dementia, but the underlying ionic and cellular mechanisms of these effects are not well understood [29-33]. Our results [28] indicate that a commonly used HCN channel blocker ZD7288 causes a complete block of hippocampal theta rhythms, which are considered to be a nonspecific memory indicator [30, 34-38]. Interestingly a limited amount of anecdotal evidence suggests that LTG may be helpful for agitation, psychosis and memory loss in patients with dementia including AD [39, 40]. According to our initial findings, it appears that LTG may counteract the detrimental effect of intracere-broventricular Aβ1-42 infusion on spontaneous theta rhythms in the hippocampus. Specifically, the data suggest that LTG is able to restore the hippocampal theta, previously suppressed by the infusion of Aβ1-42 [41]. We hypothesize a mechanistic link between HCN function, theta rhythms generation and memory formation and suggested that HCN channels could be considered as potential targets for dementia treatment. A number of underlying studies are critically reviewed here, and suggestions are given for future research.

## HCN channels alter brain function due to their physiology, structure, and distribution

### Structure and distribution of HCN channels in the brain

**Figure 1.**
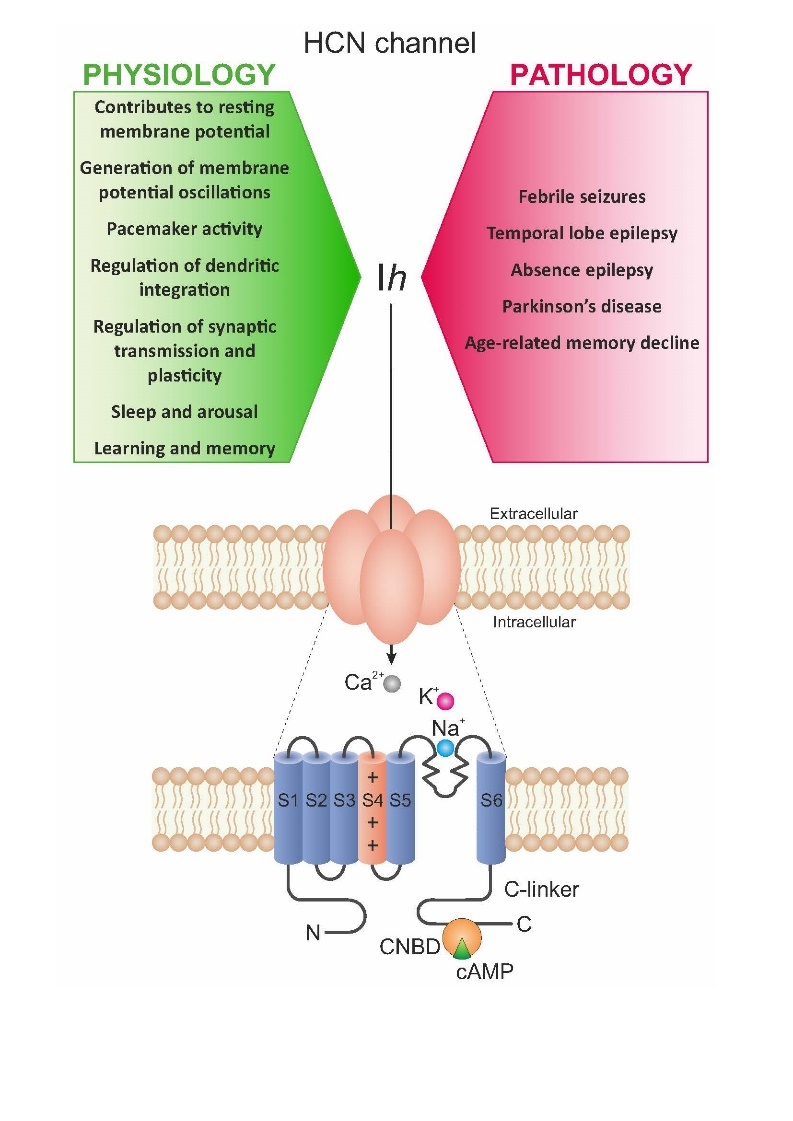
**HCN channels: functions in physiology and pathology and biochemical structure**. Based on Postea and Biel (2011).

HCN channels are voltage-gated ion channels mediating an inward current of positively charged ions activated upon membrane hyperpolarization. They regulate intrinsic excitability, pacemaker activity, and the integration of synaptic inputs. [3, 8, 21, 42, 43]. Unlike other types of voltage-gated channels, the activity of HCN channels is influenced by both membrane voltage fluctuations as well as cAMP binding. HCN channels belong to a superfamily of channels called voltage-gated pore loop channels and they are composed of four subunits (HCN1-HCN4) forming a pore allowing positive ions to pass through. Each subunit is composed of six transmembrane α-helices (S1-S6), with the positively charged helix acting as a voltage sensor (S4), loop domain between the S5 and S6 helix that forms the ion selectivity filter, and the pore region [8, 21, 42]. The C-terminal is composed of the C-linker and the cyclic nucleotide-binding domain which mediates their responses to cAMP (Fig. 1). HCN channels are formed by four subunits, which can be either all the same type (homomeric) or a combination of different subunits (heteromeric) (Fig. 1) [21].

**Figure 2.**
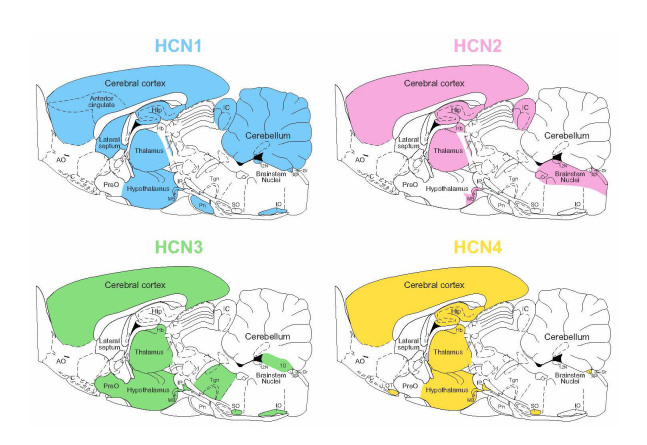
**The distribution of HCN1, HCN2, HCN3, and HCN4 channels in the rat brain**. Based on the literature review, brain regions with high expression of HCN channels were marked with colors on the representative sagittal section of the rat brain: HCN1 (blue), HCN2 (pink), HCN3 (green), and HCN4 (yellow). Abbreviations: 10 - 10^th^ cerebellum lobule, 12N - hypoglossal nucleus, AO - anterior olfactory nucleus, AP - Area postrema, fr - fasciculus retroflexus, Gr - gracile nucleus, Hb - habenula, Hip - hippocampus, IC - inferior colliculus, IO - inferior olive, IP - interpeduncular nucleus, LOT - nucleus of the lateral olfactory tract, MB - mamillary body, Pn - pontine nuclei, PreO - preoptic area, SO - supra-olivary complex, Tgn - tegmental nuclei.

**Figure 3.**
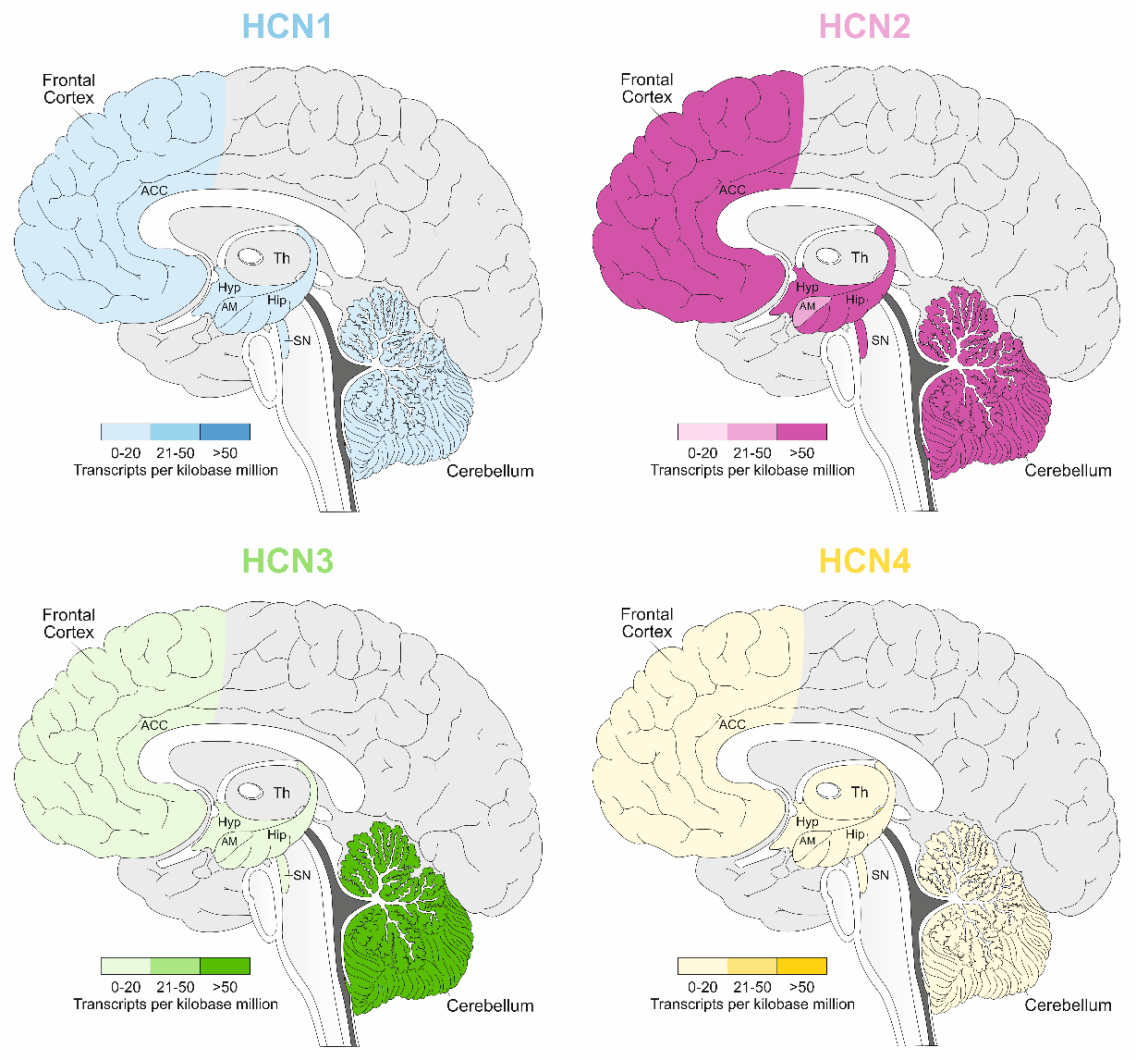
**The distribution of HCN1, HCN2, HCN3, and HCN4 channels in the human brain**. Based on the literature review, brain regions with differential expression of HCN channels were marked with colors on the representative sagittal section of the human brain: HCN1 (from light to dark blue), HCN2 (from light to dark pink), HCN3 (from light to dark green), and HCN4 (from light to dark yellow). Transcripts per kilobase million values reported for select adult human tissue samples from the GTEx Project (https://gtexportal.org). Only tissue RNA expression data corroborated by findings in the Human Protein Atlas data set and/or the FANTOM5 data set have been included (https://www.proteinatlas.org). Abbreviations: ACC - anterior cingulate cortex, AM - Amygdala, Hip - hippocampus, Hyp - hypothalamus, SN - Substantia nigra, Th - Thalamus. Based on the review from Santoro and Shah, 2020.

Each subtype of HCN channel exhibits distinct cAMP-sensitivity and displays distinct expression patterns in the nervous system [3]. All four HCN isoforms are expressed in the mammalian brain, specifically: i/ HCN1 is primarily expressed in the neocortex (it is prominent in layer 5 pyramidal neurons but not in other cortical layers), hippocampus (CA1 and CA3 pyramidal neurons and interneurons of stratum oriens and stratum lucidum), Medial Septum-Diagonal Band of Broca (MS/DBB), cerebellum and brainstem; ii/ HCN2 is scattered widely throughout brain and so it has ubiquitous nature; iii/ HCN3 shows the weakest expression and is distributed in hypothalamic nuclei, olfactory bulb, retinal cone, pedicles, MS/DBB; iiii/ HCN4 is only weakly expressed in hippocampus and neocortex but occurs in thalamic nuclei, basal ganglia, and olfactory bulb and MS/DBB [8, 42, 44-49]. A graphic representation of HCN subunit distributions in rat and human brain areas is shown in Figure 2 and Figure 3, respectively. A precise distribution of HCN 1-4 subunits in the rat and human brain with corresponding literature is shown in Table 1.

The subcellular localization of HCN channels within a cell can differ based on the type of neuron [50]. For instance, in neocortical and hippocampal pyramidal neurons HCN channels are situated in the distal branches of the dendrites. In this location, they may inhibit dendritic excitability thereby decreasing temporal integration, and decreasing the amplitude of electrical signals [3, 51-53]. In some neurons the HCN channels subunits’ expression often overlap and can assemble into different combinations. For example, in hippocampal pyramidal neurons, HCN1 and HCN2 subunits can form heteromeric complexes [54]. The variations in the expression patterns, subcellular location, and combinations of the subunits that compose HCN channels lead to diverse biophysical properties of these channels. This diversity of properties could contribute to the different roles that HCN channels play in distinct brain regions, both in normal functions as well as in pathological conditions [3].

**Table 1:** HCN1-4 subunits distribution in the rat and human brain.

HCN1-4 subunits distribution in the rat brain		
**HCN subunit**	Brain structure	Source
**HCN1**	Anterior Cingulate, Area postrema, Cerebellum, Cerebral cortex, Hippocampus (mainly CA1 pyramids), Hypoglossal nucleus, Hypothalamus, Inferior colliculus, Interior Olive, Lateral Septum Nucleus, Mamillary Body, Pontine Nuclei, Thalamus.	*Santoro et al. 2000* [49]*;* *Williams & Stuart 2000* [53]*;* *Lorincz et al. 2002* [51]*;* *Notomi & Shigemoto 2004* [48]*;* *Kuisle et al. 2006* [61]*;* *Milligan et al. 2006* [44]*;* *Varga et al. 2008* [62]*;* *Nusser 2009* [63].
**HCN2**	Brainstem Nuclei, Cerebral cortex, Gracile nucleus, External Globus Pallidus, Hippocampus, Hypoglossal nucleus, Inferior colliculus, Mamillary Body, Thalamus, The lateral habenular complex.	*Santoro et al. 2000* [49]*;* *Notomi & Shigemoto 2004* [48]*;* *Poller et al. 2011* [54].
**HCN3**	Cerebellar cortex lobule 10, Cerebral cortex, Fasciculus retroflexus, Habenular nucleus, Hypothalamus, Interior Olive, Interpeduncular nucleus, Piriform cortex, Preoptic area, Superior olivary complex, Tegmental nuclei, Thalamus.	*Santoro et al. 2000* [49]*;* *Notomi & Shigemoto 2004* [48].
**HCN4**	Area postrema, Cerebral cortex, Fasciculus retroflexus, Hypothalamus, Interpeduncular nucleus, Nucleus of the lateral olfactory tract, Superior olivary complex, Thalamus, The lateral habenular complex, Hippocampus.	*Santoro et al. 2000* [49]*;* *Notomi & Shigemoto 2004* [48]*;* *Poller et al. 2011* [54]*;* *Hughes et al. 2013* [64].
HCN1-4 subunits distribution in the human brain	
**HCN subunit**	**Brain structure** (>1 RNA transcripts per kilobase million)	**Source**
**HCN1**	Amygdala, Anterior Cingulate, Caudate, Cerebellum, Frontal cortex, Hippocampus, Hypothalamus, Substantia nigra.	*DiFrancesco and DiFrancesco 2015* [65]*;* *Santoro and Shah 2020* [66]. https://gtexportal.org https://www.proteinatlas.org
**HCN2**	Amygdala, Anterior Cingulate, Caudate, Cerebellum, Frontal cortex, Hippocampus, Hypothalamus, Substantia nigra.	*DiFrancesco and DiFrancesco 2015* [65]*;* *Santoro and Shah 2020* [66]. https://gtexportal.org https://www.proteinatlas.org
**HCN3**	Amygdala, Anterior Cingulate, Caudate, Cerebellum, Frontal cortex, Hippocampus, Hypothalamus, Substantia nigra.	*DiFrancesco and DiFrancesco 2015* [65]*;* *Santoro and Shah 2020* [66]. https://gtexportal.org https://www.proteinatlas.org
**HCN4**	Amygdala, Anterior Cingulate, Cerebellum, Frontal Cortex, Hypothalamus, Substantia Nigra, Thalamus	*Seifert et al. 1999* [67]*;* *DiFrancesco and DiFrancesco 2015* [65]*;* *Santoro and Shah 2020* [66]. https://gtexportal.org https://www.proteinatlas.org

For instance, rapidly activating HCN1 subunit is highly expressed in CA1 pyramidal neurons in hippocampus contributing to a very fast activation of the I*h* (Fig. 2) [8]. According to the study by Roth and Hu in 2020 [55], the subcellular distribution of HCN channels contributes to the high speed of synaptic inhibition mediated by parvalbumin-expressing basket cells in rats. The study revealed that distribution of functional HCN channels in these cells is exclusively limited to the axons and completely absent in somata and dendrites. A specific pattern of how HCN channels modulate the excitability of neurons depending on their developmental stage was observed in different subpopulations of interneurons located in Layer 1 of the cerebral cortex. This suggests that HCN channels may play a role in the development and ongoing function of cortical circuits by regulating the excitability of specific types of interneurons in the medial agranular Layer 1 of the cortex [56].

### I_h_ current neurophysiology and its’ link to theta rhythms generation

The I*_h_* current activates upon membrane hyperpolarization from resting potentials producing an inward, depolarizing current [8, 47, 57, 58]. I*_h_* is a mixed cation current that typically activates with hyperpolarizing steps to potentials negative to -50 to -60 mV. The HCN channel has unusual ion selectivity since it conducts both Na^+^ and K^+^ ions. The ratio of the K^+^ to Na^+^ permeability of the channel, P_K_:P_Na_, ranges from 3:1 to 5:1, yielding values for the reversal potential of -25 to -40 mV. As a consequence, activation of the channel at typical resting potentials results in a net inward current carried largely by Na^+^ [8]. An inward *Ih* current exerts two effects on the membrane: firstly, I*_h_* depolarizes the membrane and brings the membrane potential closer to firing threshold, and secondly tonic I*_h_* stabilizes resting membrane potential and reduces the membrane input resistance. This results in a suppression of membrane potential fluctuations to a given current stimulus, dampening dendritic integration and reducing synaptic-driven neuronal excitability [6, 59, 60]. As a result, I*_h_* has an exceptional ability to regulate membrane properties giving rise to a rhythmic firing, contributing to subthreshold membrane potential oscillations and dendritic integration [6, 59]. Moreover, I*_h_* maintains the membrane potential close to spike threshold, leading postsynaptic currents to integrate inputs regardless of the prior state [26, 59]. I*_h_* produces a decrease in membrane input resistance, however when the membrane is hyperpolarized, more HCN channels become opened. As a result, a slowly depolarizing inward cation current is generated, which reverses the hyperpolarization and drives the membrane potential back to its initial value. Conversely, the depolarization leads to I*_h_* deactivation, counteracting depolarization and restoring the membrane potential [3, 9, 43]. Moreover, the presence of HCN channels in the distal dendrites can modify the time course of EPSP by enhancing the local resting membrane conductance. This results in a leakage path for current flow and speeds the decay of the distal EPSP [52]. The high conductance of dendritic I*_h_*, both in neocortical neurons and CA1 neurons leads to a significant reduction of the amplitude of a distal EPSP before they reach the soma [1, 2]. Yu et al., 2004 [68] using combined whole-cell patch clamp recording and fluorescence Ca^2+^ imaging demonstrated that Ca^2+^ permeates through I*_h_* channels. The electrophysiology of HCN channels is often studied by the application of non-selective agonists and antagonists or modulators. Lamotrigine and ZD7288 are the most often used pharmacological agents modulating I*_h_* currents. It should be however pointed out that Lamotrigine also acts as a blocker of voltage-gated sodium channels and calcium channels, and reduces the activity-driven glutamate release from the presynaptic terminals of excitatory neurons [69, 70].

Lamotrigine is known to enhance I*h* current mediated by HCN channels present e.g. in pyramidal neurons through a positive shift in the voltage dependence of I*h* activation [71]. Additionally, few studies evidenced that ZD7288 has also ability to block Na^+^ channels and alter synaptic transmission [72-74]. So far, no compounds with highly selective agonist and antagonist properties for HCN channels have been synthesized.

Since activation of HCN channels in the soma generates an inward current and membrane depolarization, HCN channels might act as pacemakers to initiate spontaneous neuronal firing and contribute to rhythmogenesis and subthreshold membrane potential oscillations (MPOs) [3-5, 7, 8] sometimes referred to as the intracellular theta rhythm [75-77], contributing to the rhythmic oscillations in the brain. The role of I*_h_* current in generation of rhythmic oscillations has been acknowledged since the early 1980s in thalamocortical relay neurons [4, 78]. The ionic processes underlying electro-responsiveness of these neurons were studied using *in vitro* brain slices obtained from rodents. The after-hyperpolarization following fast spikes was markedly reduced in amplitude and duration by bath application of Cd^2+^, Co^2+^ or Mn^2+^, indicating that a large component of this response is generated by a Ca^2+^-dependent K^+^ conductance. Following hyperpolarizing current pulses, the membrane potential showed a delayed return to base line. It has been suggested that the intrinsic biophysical properties of thalamic neurons not only allow them to act as relays but also as individual cell oscillators at two specific frequencies, 9-10 and 5-6 Hz. These frequencies coincide with the alpha and theta rhythms [4]. It is currently understood that when HCN channels are activated, it leads to a depolarization of the cell membrane, which in turn activates the low-threshold depolarizing Ca^2+^ currents, lasting for tens of milliseconds and triggering a burst of action potentials. During the spike I*_h_* is deactivated, leading to a hyperpolarizing overshoot at the end of the Ca^2+^ spike. Subsequently, I*_h_* is reactivated, and the cycle regenerates [3]. A study by Kocsis and Li, 2004 [5] revealed the role of I*_h_* current in theta rhythms generation in vivo. They found that when I*_h_* current in the medial septum was blocked, it significantly reduced the frequency of hippocampal theta oscillations, without altering the context in which theta occurred in freely moving rats, e.g., during specific behaviors. Septal injection of HCN blocker ZD7288 eliminated atropine-resistant theta elicited by high intensity electrical stimulation of the reticular formation in anaesthetized rats. These findings may suggest that the functional I*_h_* current is essential for the medial septum to generate or transmit high-frequency theta elicited by ascending activation. They further indicate that the Ih current likely has a specific role in the medial septum's generation of theta rhythms, facilitating these rhythms during behaviors like exploration and rapid eye movement sleep.

It has been previously shown that I*_h_* current can act as a pacemaker current in the hippocampus and entorhinal cortex when membrane potential exceeds a certain level. [79, 80]. Sotty et al., 2003 [81] found that the I*_h_* conductance promotes neuronal burst firing, reduces the delay of rebound-firing after hyperpolarization, increases the frequency tuning of neurons, and enhances the resonance of cells, allowing them to transmit rhythmic inputs over a wide theta frequency range. A study by Hu et al., 2002 [82] demonstrated that I*_h_* current contributes to theta resonance in specific interneurons in the stratum oriens of the hippocampus. These neurons have a high density of HCN channels as previously reported [83, 84]. Theta resonance is a phenomenon in which a neuron's firing rate is maximally sensitive to inputs at a specific frequency within the theta range [81]. Spike transmission in CA1 pyramidal cells is enhanced in the theta band and this effect is due to an interaction between intrinsic cellular properties and network mechanisms. Pharmacological blockade of I*_h_* abolished theta resonance in CA1 pyramidal cells [85]. Nolan et al., 2004 [10] observed that deletion of HCN1 causes a general enhancement in the voltage response to low-frequency oscillatory currents, consistent with the enhancement in theta power. According to Sotty et al. in 2003 [81], I*_h_* current present in the medial septal neurons is more likely to support resonance than pacemaker activity. This is because the presence of I*_h_* allows the modulation of theta frequency in accordance with the level of input signals ascending from the brainstem. Additionally, I*_h_* was found in bursting GABAergic neurons of the MS/DBB, which is considered to be a higher-level relay/generator of theta rhythms [5, 50, 81, 86, 87]. According to a study by Xu et al. in 2004 [50], when HCN channels were selectively blocked by ZD7288, the reduction of spontaneous firing of septohippocampal GABA-ergic neurons in rat brain slices was observed. Additionally, when ZD7288 was locally infused into the MS/DBB region, it decreased exploratory behavior and hippocampal theta bursts evoked by sensory stimuli in behaving rats [50]. Therefore, the authors proposed that I*_h_* in septo-hippocampal GABAergic neurons contributes to the hippocampal theta rhythms generation. Our findings support this, since intrahippocampal injections of HCN modulator - LTG significantly increased theta amplitude *in vivo* while ZD7288 caused a complete EEG silence *in vivo* as well in hippocampal acute slices [28]. It should be noted, however, that Lamotrigine has the ability to block Na^+^ channels which may also contribute to the observed effect.

A study by Stadler et al., 2014 [88] evidenced that elevation of interferons by viral brain infection causes a reduction in the I*_h_* current in cortical pyramidal neurons. When rodent brain slices were directly exposed to type I interferons, the HCN1 subunit was specifically affected. This reduction in I*_h_* current resulted in hyperpolarization of the resting membrane potential, shift in the resonance frequency, and increase in membrane impedance of interneurons. When interferon-β was infused into the cerebral cortex of rodents in vivo, it reduced the power of higher frequencies in the EEG activity, but only in the presence of HCN1. Das and Narayanan, 2017 [89] measured the spike-triggered average (STA) of rat hippocampal pyramidal neurons and quantified spectral selectivity in their spike initiation dynamics and their coincidence detection window. These authors revealed a strong theta-frequency selectivity in the STA as well as found that the STA resting frequency was significantly reduced after HCN channel blockade; thus, expanding a role of HCN channels to control theta-frequency selectivity in spike initiation dynamics.

### Ih current and memory formation

Increasing evidence has implicated I*_h_* in dendritic integration, activity-dependent plasticity, learning and memory, sleep and arousal [3, 6, 10]. Dendritic HCN channels may modify the integrative properties of neurons normalizing effects of multiple inputs [52, 90]. HCN channel distribution to certain sites on dendrites play a key role in local dendritic processing while their density may control temporal summation properties [91], this could be vital for understanding the role of I*_h_* in memory formation [6].

Using generalized and regional knockout mice, Nolan et al., 2003 [26] discovered that deletion of the HCN1 channel caused profound motor learning and memory deficits. This suggests that HCN1 channels play a critical role in cognitive processes. HCN1 channels were found to mediate an inward current which helps to stabilize the integrative properties of Purkinje cells and ensures that the input-output function of these cells is not affected by their previous activity. The authors of the study claimed that the non-synaptic integrative function of HCN1 subunits is essential for precise decoding of input patterns, enabling synaptic plasticity to appropriately modulate the performance of the motor activity. Subsequently, Nolan et al., 2004 [10] conducted a study using mice with general or forebrain-restricted knockout of the HCN1 gene to investigate the role of HCN1 channels in the development of spatial memory and plasticity in CA1 pyramidal neurons. Interestingly, they found that the absence of HCN1 channels resulted in significant improvement of theta oscillations, enhancement in spatial memory, and long-term potentiation (LTP) at the direct perforant path input to the distal dendrites of CA1 pyramidal neurons. The authors proposed that HCN1 channels constrain learning and memory by regulating dendritic integration of distal synaptic inputs to pyramidal cells [10]. In a later study, the same authors suggested that HCN1 channels expressed by stellate neurons in layer II of the entorhinal cortex play a crucial role in processing inputs to the dentate gyrus of the hippocampus [9]. Interestingly, HCN channels are abundant in layer V of the neocortex and in CA1 pyramidal neurons, where Ih serves to dampen temporal summation of multiple synaptic inputs at the soma and increases bidirectional attenuation of EPSPs [1, 53]. Phillips et al., 2014 [20] proposed that a post-spike-and-wave discharge change in HCN1 transcript levels is a useful biomarker of hippocampal plasticity, as previously summarized memory deficits may be due to I*h* up- and downregulation [3, 6]. This can occur in prefrontal cortex and has been associated with age-related memory decline. Wang et al., 2007 [11] in electrophysiological studies evidenced that, either α2A-AR stimulation, cAMP inhibition or HCN channel blockade enhanced spatially tuned delay-related firing of neurons in prefrontal cortex. In their behavioral studies, either blockade or knockdown of HCN1 channels in prefrontal cortical neurons improved water maze performance. More recent studies have shown that cannabinoid type-1 receptors (CB1Rs) control hippocampal synaptic plasticity and spatial memory through HCN channels [92]. Additionally, a study by Stieglitz et al. in 2018 [93] showed that the HCN3-knockout mice demonstrated impaired processing of contextual information. This was characterized by a weakened long-term extinction of contextual fear and an increased fear response to a neutral stimulus upon repeated exposure. Furthermore, the function and expression of HCN1 channels are changed during the development of depression and a reduction of HCN1 protein expression may affect the resilience to chronic stress [94].

## Theta rhythms: a facilitating role in memory formation

The first researchers reporting regular slow wave activity in mammals were Jung and Kornmuller, who in 1938 [95] registered theta oscillations in the hippocampus of rabbits. Green and Arduini, 16 years later [96], conducted even more comprehensive research on the hippocampal theta rhythms and its’ modification by several receptor systems in rabbits, cats and monkeys. Since then, researchers studying this newly described phenomenon have divided into two "camps" using separate cognitive strategies. One was to find a correlation between theta rhythms and various behavioral and mental states in mammals [96-104] while the goal of a second stream of research was to study the physiology of theta *per se*. The latter focused on the study of the hippocampal theta rhythms topography, cellular mechanisms and neurochemical processes involved in its generation [97, 105-109]. It is now widely accepted that the theta rhythms observed in the mammalian limbic cortex are a prime example of a field rhythmic oscillatory pattern based on central mechanisms of synchronization. These rhythms are thought to play an important role in a variety of cognitive processes, including memory and attention [97, 106, 110, 111]. Moreover, theta rhythms constitute a key physiological phenotypic property that may serve as a sensitive assay enabling the study of neural network excitability [110]. Theta activity is a sinusoidal, high-voltage (from 0.2 to 2 mV, extracellular) oscillatory pattern with a frequency range from 3 to 12 Hz in rodents. [97, 106, 111]. Studies of Leblanc and Bland, 1979 [112] on the ontogenesis of theta rhythmic activity in rodents have shown that this pattern appears around the 10’th day of postnatal development and then its frequency and amplitude increase for the next two weeks, until typical values for an adult animal are reached. Theta oscillations also occur in primates including humans, in whom it reaches a frequency of 4 to 8 Hz [113-115].

Lopes da Silva in his extensive review described three basic functions of theta rhythms occurring in the limbic cortex: i/ gating function for the information flow within the hippocampal neuronal network, ii/ enabling transmission of information from hippocampus to higher cortical structures, iii/ involvement in LTP generation [116]. These suppositions found a detailed explanation in later studies on the functional significance of theta rhythms and today the role of hippocampal theta in learning and memory formation is indisputable [34-38]. According to some authors, theta rhythms are a form of communication between neurons, leading to the establishment of certain discharge patterns in a neural network [117-119]. Rodent EEG recordings have shown that during goal-directed behaviors, neurons in prefrontal cortex increase the degree of synchronization in theta frequency ranges, thus creating a state of "hyper-synchronization" or consistency with the hippocampal rhythm [120-122]. Theta rhythms also occur during REM sleep and may be involved in forming memory traces by inducing LTP in hippocampus [98, 123, 124]. The hippocampal theta is present in the EEG while the animal performs locomotor activities, such as arbitrary movements, orientation reflexes, exploratory and preparatory movements or running [99, 102, 104, 124]. It appears in the hippocampal field recordings during behavior involving reception of sensory stimuli such as the sense of smell [125]. Experiments carried out on rats provide information on the role of hippocampal theta rhythms in planning and initiation of motor sequences [126]. The hippocampal theta is also involved in the regulation of emotional behavior, since it regulates memory-anxiety interactions affecting the ability to make decisions in conflict situations [127]. Findings by Sakimoto and Sakata, 2020 [128] provide strong support for the assumption of the conflict resolution model that the role of the hippocampal theta in memory formation is to inhibit responses to conflicting stimuli during non-spatial stimulus discrimination tasks.

The most prominent evidence for the involvement of hippocampal neural networks in memory formation in humans comes from 1957, when William Beecher Scoville and Brenda Milner performed a bilateral hippocampal lesion in Henry Molaison a patient suffering from frequent epileptic seizures. The surgery resulted in complete post-surgical amnesia and mild retrograde amnesia, and the patient suffered from severe damage to long-term memory and declarative memory [129]. Today it is accepted that hippocampal theta coordinates memory processing in humans [130-132]. Research by Kaplan et al., 2012 [115] shows that hippocampal theta rhythms in humans serves as a pattern strengthening memory process by coordinating exploratory movements. Fell et al., 2003 [131] discovered that rhinal-hippocampal theta coherence might be associated with slowly modulated coupling related to an encoding state during declarative memory formation. A role of hippocampal theta rhythms in attention processing in patients subjected to virtual navigation paradigms have also been demonstrated [133]. A significant relationship has also been shown between hippocampal theta and the degree of spatial memory development in humans [134, 135]. This is probably related to the activity of "place cells" that discharge when an individual is in a specific place in space [136]. Theta rhythms present in hippocampus play an important role in remembering the sequence of specific events and storing the contextual relationships between individual stimuli [130]. Lega et al., 2012 [137] performed intracranial EEG (iEEG) recordings from electrodes placed in the hippocampal area of 33 neurosurgical patients as they performed an episodic memory task. They identified two patterns of rhythmic oscillations in the hippocampus, at ~3 (slow-theta) and ~8 Hz (fast-theta). They found that the slow-theta oscillations generated in the human hippocampus resembled the memory-related theta oscillations observed in animals. Additionally, they found that both fast- and slow-theta rhythms exhibited phase synchronization with oscillations in the temporal cortex. Some recent human studies have also suggested that memory retrieval would be superior following theta-burst stimulation. Healthy adults were administered four different single-session stimulation conditions to the same parietal cortex location of the hippocampal-cortical network and measured the aftereffects on fMRI connectivity and episodic memory retrieval. Continuous theta-burst stimulation improved item retrieval success relative to sham and relative to beta-frequency stimulation [138]. Kota et al., 2020 [139] used an associative recognition memory procedure to identify hippocampal correlates of successful associative memory encoding and retrieval in patients undergoing intracranial EEG monitoring. Their results provided direct electrophysiological evidence that 2-5 Hz hippocampal theta oscillations preferentially support the formation of associative memories. They also found that the reinstatement of rhythmic patterns in the hippocampus was stronger for successful memory recollection. Herweg et al., 2020 [140] re-evaluated human studies and mixed evidence for theta’s role in learning to conclude that successful memory is associated with increased narrow-band theta oscillations and that theta specifically supports associative memory and the retrieval of episodic memories. Johnson et al., 2022 [141] conducted a study that used rare direct electrophysiological recordings from children and adolescents to investigate how memory is linked to the interactions between the medial temporal lobe (MTL) and prefrontal cortex (PFC). They discovered that the MTL and PFC interact through two different theta mechanisms: a slow oscillation, at around 3 Hz, that supports amplitude coupling and slows down with age, and a fast oscillation, at around 7 Hz, that supports phase coupling and speeds up with age. The interactions between these slow and fast theta rhythms immediately preceding scene onset were found to explain age-related differences in recognition performance. These findings provided insights into the system-level dynamics of memory formation. Ratcliffe et al., 2022 [142] obtained high-density EEG data from fronto-medial cortex while participants engaged in working memory-dependent tasks to demonstrate that frontal theta orchestrated posterior maintenance of working memory content. Griffiths et al., 2021 [143] found that hippocampal theta/gamma phase-amplitude coupling, increased during mnemonic binding (but not sequence perception) and correlated with enhanced memory performance. They suggested that hippocampal theta-gamma phase-amplitude coupling could support the binding of information into a coherent memory trace.

Other authors recorded single-neuron and local field potentials from the human hippocampus in epilepsy patients implanted with depth electrodes [144]. They recorded theta rhythms while patients performed a visual recognition memory task. They also found that human theta appeared in short oscillatory bouts whose properties varied between hemispheres [144]. Klimesch, 1999 [145] in his extensive review suggested that the encoding of new information is reflected by theta oscillations in hippocampo-cortical feedback loops, whereas search and retrieval processes in (semantic) long-term memory are reflected by upper alpha oscillations in thalamo-cortical feedback loops. Buzsaki and Moser, 2013 [146] hypothesized how specific firing patterns and oscillatory dynamics in the entorhinal cortex and hippocampus support both navigation and memory.

The occurrence of synchronous field activity in theta band depends on intracellular biophysical phenomenon involving the repetitive rhythmic oscillations of membrane potential (MPOs) [75, 76, 147]. MPOs are often defined as an intracellular theta rhythm and occur in close correlation with the phase of the extracellularly recorded theta, and disappear when theta oscillations are absent [75-77]. So far, it has not been possible to clearly state what mechanisms are involved in generating MPOs. Some authors point to the participation of IPSPs [148, 149] while others suggest the involvement of EPSPs [77, 150, 151]. It seems highly probable that the appearance of MPOs is the result of the specific, inherent, properties of neuronal cell membranes [75, 76, 106, 152]. Some authors suggest that voltage-gated sodium and calcium channels [106] or HCN channels may be responsible for MPOs [3-5, 7, 8, 147]. Another mechanism that could be responsible for theta rhythms generation is ephaptic coupling, in which depolarization waves flow in all directions, recruiting nearby neurons to also discharge rhythmically [106]. Turner et al., 1984 [153] suggested that ephaptic interactions may potentiate the extracellular population spikes by recruiting subthreshold neurons within the population during repetitive afferent stimulation. It is well established that neurons may be ephaptically coupled to the frequencies of the local field potential, particularly theta, which can lead to effective neuronal synchronization, however the exact mechanism for this is largely unknown [154-156]. The appearance of hippocampal theta may also depend on the relationship between synaptic inputs and neuronal intrinsic electrical properties, such as resonance, relaying on the ability of neurons to respond to inputs at specific, preferred frequencies [6, 82, 157]. Interestingly, activity-dependent LTP caused an increase in resonance frequency, which was observed at more distal sites on the dendrites of hippocampal neurons along with increased Ih density [158]. Therefore, it is highly probable that the intrinsic resonant properties of pyramidal neurons could be substantially modified by the deletion of HCN subunits changing the parameters of hippocampal theta such as an enhancement of power, that was observed by Nolan et al. (2004) [10]. Ephaptic coupling and resonance could be the mechanistic link between HCN properties and appearance of theta oscillations contributing to changes in memory formation [6]. In addition to their contribution to cell resonance, the HCN channels have a number of properties that suggest they might play a major role in control of oscillations in hippocampus and other brain areas. Previous research has indicated that HCN channels, particularly the HCN1 subtype, play a crucial role in the modulation of hippocampal-based memory [10, 159]. Microiontophoretic blockade of HCN channels resulted in the reduction of discharge frequency and perturbation of theta frequency firing [62]. A multiscale computer-based model demonstrated that modulation of pyramidal and basket cell I*h* currents allows tuning theta and gamma oscillation frequency and amplitude [160].

## HCN dysregulation and disruption in age-related memory decline and dementia including AD

**Figure 4.**
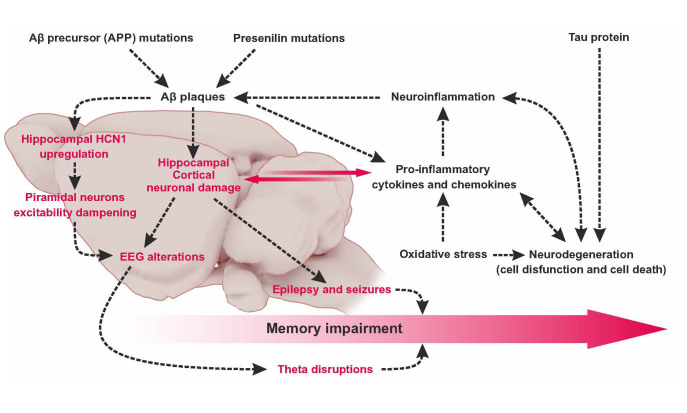
**The proposed vicious cycle of toxicity and neuroinflammation leading to neuronal dysfunction and neurodegeneration in the hippocampus and the neocortex manifested as EEG alterations such as theta disruptions, epilepsy and seizures in the course of dementia in AD development**. Oxidative stress contributes to Aβ generation through inflammation triggered by oxidative conditions, mainly including the releasing of proinflammatory cytokines and chemokines. Changes in hippocampal and/or cortical oscillatory activity might precede Aβ induced epileptiform activity and correlate with the alterations in memory processes in AD/dementia development.

Dementias, including Alzheimer's disease, are neurodegenerative disorders characterized by memory impairment and diminished cognitive performance [161]. Non-cognitive neurological conditions often occurring alongside include symptoms such as depression, aggression, and psychosis [162]. In case of AD progression an early breakdown of neurotransmission, particularly in the cholinergic system, is often observed [163], as well as, neuroinflammation [164], and loss of white matter [165]. The accumulation of the β-amyloid peptide (Aβ), being still discussed as a hallmark of AD, mainly affects the hippocampal formation, associative cortices, and some subcortical structures [166] leading to neurodegeneration, membrane disruption, and synaptic dysfunction (Fig. 2) [167, 168]. Aβ is derived by proteolytic cleavage from the amyloid precursor protein (APP) by two proteolytic enzymes: β-secretase (beta-site APP cleavage enzyme, BACE1) and γ-secretase [169, 170]. TNF-α as a main inflammatory cytokine produced by macrophages/monocytes during acute inflammation [171] may directly stimulate BACE1 expression and enhance β- processing of APP in astrocytes [172]. The other hallmark often suggested in pathology of human AD is the intraneuronal aggregation of hyperphosphorylated tau protein known as neurofibrillary tangles deposits [173]. Also, mutations in the PSEN1 gene, encoding presenilin-1 (PS1), are thought as the most common cause of familiar Alzheimer’s disease (FAD) since PS1 functions as the catalytic subunit of γ-secretase [161]. Thus, Aβ deposition and tau accumulation may be associated with dementia in AD development, whereas various mechanisms accompanying neuronal degeneration and dysfunction have been proposed, which include, oxidative stress and inflammatory processes [33, 174-176], genetic factors [177], and environmental impact factors (Fig. 4) [178, 179]. The real facet of AD onset and progression has been gradually revealed after decades of studies not only about Aβ deposition, tau accumulation and neuronal interactions but also about more profound understandings of glial interactions [180]. Interestingly in recent years early evidence has been accumulating that anti-tau interventions similarly to anti-Aβ approaches do not work in tauopathies and AD treatment [181]. It is also clear that oxidative stress and damage to mitochondrial DNA associated with aging can impair mitochondrial energy metabolism and ion homeostasis in neurons, thereby rendering them vulnerable to degeneration [33]. Interestingly oxidative stress enhances both BACE1 and γ-secretase activity and toxic Aβ formation as well as tau hyperphosphorylation [182]. Additionally, neuroinflammation is a common feature of age-related neurodegenerative diseases [176]. Previous reports evidenced that interaction between oxidative stress and neuroinflammation leads to dementias and AD development [174, 175, 183, 184]. An important feature of the hypothesis of AD and dementias pathology is that oxidative stress may trigger selfperpetuating cycle of chronic neuroinflammation, which serves to further promote oxidative stress and may contribute to irreversible neuronal dysfunction and cell death [185]. Described factors may interact and amplify each other in a vicious cycle of toxicity, leading to neuronal dysfunction and finally cell death in the hippocampus, the neocortex, and several other structures of the brain [186], which was illustrated in Fig.4. Luo et al., 2016 [187] found evidence to support the idea that AD and age-related dementia may have mixed pathology using a multiple familial mouse models and a novel sporadic model of AD. They have observed that Aβ accumulation primarily affects the hippocampus, associative cortices, and certain subcortical structures [166]. Since HCN channels are widely distributed in these regions they may participate in the etiology of dementias including AD by affecting neuronal excitability [22, 188, 189] and possibly oscillatory activity including theta rhythms.

There is some limited evidence suggesting that certain anticonvulsant drugs, including some HCN modulators, like Lamotrigine, may be effective in reducing agitation, psychosis, and memory loss in older individuals with dementia, including Alzheimer's disease [39, 40]. Lamotrigine has been shown to be effective in treating other conditions such as Amyotrophic Lateral Sclerosis and Parkinson's disease in clinical settings [190]. Lamotrigine has a well-established safety profile and has been shown to be effective in preventing mood episodes from recurring in patients with bipolar disorder, in addition, none of the patients had discontinue LTG due to side effects [40, 71, 191]. LTG is well absorbed after oral administration and its bioavailability is ~98%. When used in 11 patients with the diagnosis of probable AD, LTG improved word recognition, naming and improved mood on the Alzheimer Disease Assessment Scale (ADAS) [40]. It has long been known that Lamotrigine depresses voltage sensitive sodium channels that appear to be upstream of glutamate release sites in nerve terminals [69]. Postsynaptic glutamate AMPA receptors are also inhibited by LTG [192]. Recent evidence indicates that it may also affect other neurotransmitter systems as well [28, 193]. Some new clinical applications are emerging for Lamotrigine, including: nasal formulations for rapid treatment in epilepsy [194] and a treatment for Lennox-Gastuat syndrome [195].

In animal studies it was found that LTG suppressed the induction of Aβ1-42 in the hippocampus of ischemic rats and restored cognitive function of rats subjected to 60 min of carotid occlusion, including enhanced spatial learning and memory ability. Lamotrigine has been shown to significantly reduce damage to pyramidal neurons caused by ischemia [20]. Additionally, LTG rescued the electrophysiological and cognitive deficits in two independent Neurofibromatosis type 1 mouse models [196]. Since the mechanisms underlying reduction of Aβ by LTG treatment remain unclear, Wu et al., 2015 [197] investigated the effect of LTG treatment on BACE1 protein and mRNA levels. Additionally, they described the activation of autophagy by LTG, which was accompanied by inhibition of the mammalian target of rapamycin (mTOR) signaling and activation of cAMP response element binding protein (CREB). In addition, LTG treatment reduced protein levels of BACE1 (but not mRNA levels) through activation of autophagy [197]. Interestingly, it has been found that, in a mouse model of epilepsy, HCN channelopathy is a potential link of epileptic seizures and Aβ generation. Results obtained on mice devoid of HCN1 channels show that reduced activity of I*h* current resulted in enhanced neuronal excitability, which increased seizure susceptibility and Aβ generation [17, 22]. Additionally, it has been observed that HCN1 levels drastically decrease in the temporal lobe of cynomolgus monkeys as they age, and are significantly lower in the temporal lobe of patients with sporadic AD [22]. Because HCN1 associates with APP and X11 or X11L in the brain [191], genetic deficiency of X11/X11L may induce aberrant HCN1 distribution along with epilepsy. Voltage-clamp recordings showed that I_h_ current was significantly increased in neurons from Aβ-treated rats, also confirmed by showing upregulation of the mRNA of HCN1 channels in the CA1 pyramidal layer of hippocampus (Fig. 4) [198]. To examine potential biophysical changes in the hippocampus linked to age-related AD pathogenesis on patch-clamp electro-physiology Russo et al., 2021 [199] investigated amyloid plaque deposition along the dorsoventral axis in two strains of transgenic AD (ADTg) mouse models. They found dorsoventral differences in amyloid load in aged ADTg mice as well as subthreshold physiological changes in ventral CA1 pyramidal neurons indicative of HCN channelopathy. The authors evidenced that HCN channels are functionally linked to tau abnormalities. Alterations in HCN channel expression were detected in both Tau35 mouse and human post-mortem AD brain by Goniotaki et al., 2021 [200]. Tau35 neurons exhibited altered synaptic cytoarchitecture, including progressive reductions in dendritic branching, pre-synaptic vesicles, spine density and synaptic markers, along with the development of tau pathology. These changes were accompanied by functional abnormalities in network activity, including increased HCN-dependent sag voltage. Anatomical studies in both human subjects with AD and rhesus macaques have revealed that the first signs of tau pathology appear in the stellate cell islands located in entorhinal cortex (ERC) layer II [201]. The authors used high-spatial resolution immunoEM in order to localize HCN1 subunits in young rhesus macaque ERC layer II. They suggested that HCN1 subunits are positioned to provide a signature of flexibility in postsynaptic compartments in ERC layer II stellate cells, which becomes a signature of vulnerability when abrogated by advancing age. The latest evidence from animal studies indicates that LTG restores electrophysiological alterations, prevents memory deficits, and counteracts the increase in extracellular Aβ induced by seizures in pre-symptomatic Tg2576 mice -a transgenic model of AD [216]. Similarly, Fu et al. (2023) [202] have shown that LTG substantially improved spatial cognitive deficits of APP/PS1 mice; alleviated damage to synapses and nerve cells in the brain; and reduced amyloid-β levels, tau protein hyperphosphorylation, and inflammatory responses.

Due to literature discrepancies and a scarcity of data, it remains unclear whether HCN channel modulators such as Lamotrigine could improve treatment of neurological disorders such as memory decline and dementia. Nevertheless, HCN, channels are proposed to play an important role in the molecular linkage between epileptic seizures and Aβ generation, and in the aggravation of sporadic AD [22].

## Theta rhythms alterations in dementias including AD

As stated earlier, theta rhythms appear in physiological states but also can be considered as non-specific markers of neurological disorders. Some authors have suggested that subtle changes in theta and gamma rhythms occur during the very early stages of AD and could be used as a possible predictor for the disease [30, 203]. A disruption of oscillatory network activity has been detected in the EEG of AD patients [31] and transgenic AD animals [204]. Mugantseva and Podolski, 2009 [33] showed that a decrease in low-frequency theta band oscillations and the weakening of binding between the dorsal hippocampus and the frontal cortex under the action of Аβ25-35 may underlie the typical memory breakdown associated with AD. Intrahippocampal injection of Aβ 1-42 two weeks before EEG recordings dramatically slowed hippocampal and cortical baseline EEGs [205]. Interestingly, Sun and Alkon, 2002 [206] have shown that intracere-broventricular application of Aβ in rats impaired learning and memory 3 days after injection, which was associated with a failure of hippocampal neurons to produce MPOs upon carbachol application *in vitro* [206]. Wirt et al., 2021 [207] found that animals with Streptozotocin (STZ)- induced AD model had specific changes in hippocampal and cortical network activity. They observed distinct patterns of network changes in both the anterior cingulate cortex (ACC) and hippocampus, as well as hypersynchrony within and between these regions. According to our recent study, direct intracere-broventricular Aβ1-42 injections cause a significant decrease in the amplitude and power of hippocampal theta after 7, 14, and 21 days from the injection in rats. Moreover, acute intrahippocampal injections of Lamotrigine increased the level of theta synchrony in Aβ1-42 treated animals [41]. Interesting animal studies performed by Chen et al., 2021 [208] have revealed novel findings on how gamma oscillations are affected in the olfactory bulb of mice with AD model. They found that alterations in gamma rhythms and GABA-signaling levels were present in 3-5-month-old AD mice, representing early signs of AD pathogenesis.

Recent human studies suggest that theta rhythms could be considered as a potential early biomarker of cognitive decline in AD patients [209-211]. Following the lead that the power of quantitative EEG could be used as a potential diagnostic tool for dementia in AD, Musaeus et al., 2018 [211] found a visible increase in global theta range and a decrease in high frequency power in the temporal regions for AD patients when their eyes were closed. The relative theta power was linked to multiple neuropsychological measures and had the strongest correlation coefficient with total tau. These findings indicate that the relative theta power increase may be the first change observed in patients with dementia due to AD progression. Results obtained by Goodman et al., 2018 [210] provide evidence for a relationship between altered theta-gamma coupling and working memory deficits in AD patients and mild cognitive impairment. Similarly, Spinelli et al., 2022 [203] provide the first longitudinal evidence on the impact of brain amyloidosis on the EEG dynamics of a large-scale, monocentric AD cohort. They show that different neural markers are in play at different time points of the follow-up. Theta band power increase seems to play a crucial role in pre-state of AD. Schumacher et al., 2020 [212] investigated the differences in quantitative EEG measures between highly phenotyped patients with mild cognitive impairment with Lewy bodies vs. mild cognitive impairment in AD in comparison with similarly aged healthy controls. They found there was a shift in power from beta and alpha frequency bands towards pre-alpha and theta range in patients with mild cognitive impairment with Lewy bodies which was not characteristic for AD patients. Interestingly, accelerated, intermittent theta-burst stimulation of the dorsolateral prefrontal cortex appeared to be an effective, and well-tolerated complementary treatment for AD patients [213]. A promising biomarker may be derived from the frequency analysis of electroencephalographic oscillatory responses during cognitive demands as evidenced by Yıldırım et al., 2021 [214] in patients with dementia with Lewy bodies and Parkinson's disease dementia. The study showed that electroencephalographic oscillatory responses in theta power and phase-locking during a standard visual oddball paradigm probing visual focused attention and short-term memory may be suitable biomarkers to investigate working cognitive brain systems in both groups of patients. Results obtained by Perez et al., 2022 [215] confirm previous evidence showing that older people with subjective memory complaints are characterized by distinct power resting state EEG rhythms, especially at increased theta power. These authors propose that electrophysiological biomarkers of brain dysfunction may identify cognitive decline before they are observed in a neuropsychological assessment.

## Conclusions and future perspectives

In this review, we provide a summary of the recent evidence regarding the pharmacological potential of HCN channel modulators, specifically Lamotrigine, for the supplementary treatment of major neurocognitive disorders, including AD. Additionally, we discuss the emerging evidence suggesting that theta rhythms could serve as an early biomarker for cognitive decline in patients with AD and other types of dementia.

The key concept we propose is that the activity of HCN channels, which regulate I*h* current and modulate membrane oscillations and neuronal excitability, directly influences the power and frequency of theta oscillations in brain regions involved in memory processing, such as the hippocampus and prefrontal cortex. Moreover, we emphasize the role of proinflammatory processes and the accumulation of Aβ plaques and tau depositions in the development of dementia. These factors contribute to neuronal damage in the brain, rendering HCN channels vulnerable and ultimately leading to disturbances in theta rhythms.

Lamotrigine, a non-selective enhancer of Ih current, has been shown to suppress the induction of Aβ1-42 in the hippocampus of ischemic rats and restore cognitive function. It activates autophagy, inhibits mTOR signaling, and reduces BACE1 expression at the protein level [197]. Studies on mice with an HCN1 knockout have demonstrated that a reduction in *Ih* current leads to enhanced neuronal excitability, increasing seizure susceptibility and Aβ generation [17, 22]. HCN1 levels decrease significantly in the temporal lobe of aging cynomolgus monkeys and patients with sporadic AD [22]. The expression of HCN channels has been found to be altered in post-mortem brains of AD patients, suggesting their potential involvement in the pathogenesis of the disease [200]. There is emerging evidence suggesting a potential link between biophysical changes in hippocampal neurons, amyloid plaque deposition, and the functional role of HCN channels in tau abnormalities.

Theta waves have been proposed as potential electrophysiological biomarkers for neurocognitive impairments, allowing early identification before neuropsychological assessments [209-211, 215]. However, the therapeutic efficacy of HCN channel agonists in memory decline and dementia, along with their impact on theta parameters, remains unclear due to discrepancies in the literature and limited data. Further research is needed to elucidate the precise mechanisms underlying HCN channels' involvement in amyloid plaque deposition and tau abnormalities, and to explore the therapeutic potential of HCN channel modulation in mild and major neurocognitive disorders.

In summary, Lamotrigine exhibits promising effects in suppressing Aβ induction and restoring cognitive function in animal models. Altered HCN channel expression and activity in AD and aging highlight their potential role in disease progression. Theta waves hold promise as early biomarkers, but further investigation is necessary to determine their diagnostic value. Future studies should focus on unraveling the molecular mechanisms connecting HCN channels, amyloid plaque deposition, and tau abnormalities, and evaluating the therapeutic benefits of HCN channel modulation in memory decline and dementia.

To address unresolved issues and advance the field forward, future research efforts could be directed towards the following areas:

- Synthesis of more selective HCN-openers/agonists or analogs of Lamotrigine to investigate their potential as pharmacological treatments for dementias, including AD.
- Investigating the mechanisms of action of Lamotrigine through diverse, combined approaches, including knockout mouse models, electrophysiological recordings, and molecular biology tools, to gain valuable insights into how LTG influences neuronal physiology in memory-related brain regions of both healthy and aging individuals.
- Understanding the neuroprotective and anti-inflammatory properties of Lamotrigine through comprehensive studies using specific cell lines and molecular biology techniques.
- Conducting comprehensive human studies to reveal the relationships between early changes in brain regions affected by neurodegeneration and inflammation in dementias, including AD, disruptions in I*h* current, theta oscillation alterations, and cognitive decline.

By addressing these research areas, we can gain a deeper understanding of the potential therapeutic benefits of HCN channel modulators, such as Lamotrigine, in the treatment of dementia. Additionally, it will shed light on the underlying mechanisms involved and help pave the way for the development of more effective interventions for memory decline in mild and major neurocognitive disorders.
